# Green Synthesis and Characterization of Cobalt Oxide Nanoparticles Using *Psidium guajava* Leaves Extracts and Their Photocatalytic and Biological Activities

**DOI:** 10.3390/molecules27175646

**Published:** 2022-09-01

**Authors:** Rajakumar Govindasamy, Vaishnavi Raja, Sonalika Singh, Mydhili Govindarasu, Sulthana Sabura, Kaliaperumal Rekha, V. Devi Rajeswari, Salman S. Alharthi, Manju Vaiyapuri, Rajagopal Sudarmani, S. Jesurani, Baskar Venkidasamy, Muthu Thiruvengadam

**Affiliations:** 1 Department of Orthodontics, Saveetha Institute of Medical and Technical Sciences (SIMATS), Saveetha Dental College and Hospitals, Saveetha University, Chennai 600077, Tamil Nadu, India; 2 PG and Research Center of Physics (JAC), Mother Teresa Women’s University, Kodaikanal 624101, Tamil Nadu, India; 3 Institute of Allied Medical Science and Technology, NIMS University, Jaipur 303121, Rajasthan, India; 4 Biomedical Research Unit & Laboratory Animal Centre, Department of Anatomy, Saveetha Institute of Medical and Technical Sciences, Saveetha Dental College and Hospitals, Saveetha University, Chennai 600077, Tamil Nadu, India; 5 Department of Environmental and Herbal Science, Tamil University, Thanjavur 613005, Tamil Nadu, India; 6 Department of Biomedical Sciences, School of Biosciences and Technology, VIT University, Vellore 632014, Tamil Nadu, India; 7 Department of Chemistry, College of Science, Taif University, P.O. Box 110999, Taif 21944, Saudi Arabia; 8 Molecular Oncology Lab, Department of Biochemistry, Periyar University, Salem 636011, Tamil Nadu, India; 9 School of Engineering, Avinashilingam Institute for Home Science and Higher Education for Women, Coimbatore 641108, Tamil Nadu, India; 10 PG and Research Center of Physics, Jayaraj Annapackiam College for Women, Periyakulam 625601, Tamil Nadu, India; 11 Department of Oral and Maxillofacial Surgery, Saveetha Dental College and Hospitals, Saveetha University, Chennai 600077, Tamil Nadu, India; 12 Department of Crop Science, College of Sanghuh Life Sciences, Konkuk University, Seoul 05029, Korea

**Keywords:** *Psidium guajava*, Co_3_O_4_NPs, XRD, EDAX, cytotoxicity, photocatalytic activity, MCF-7, HCT 116 cells

## Abstract

The advanced technology for synthesizing nanoparticles utilizes natural resources in an environmentally friendly manner. Additionally, green synthesis is preferred to chemical and physical synthesis because it takes less time and effort. The green synthesis of cobalt oxide nanoparticles has recently risen due to its physico-chemical properties. In this study, many functional groups present in *Psidium guajava* leaf extracts are used to stabilize the synthesis of cobalt oxide nanoparticles. The biosynthesized cobalt oxide nanoparticles were investigated using UV-visible spectroscopic analysis. Additionally, Fourier-transform infrared spectroscopy revealed the presence of carboxylic acids, hydroxyl groups, aromatic amines, alcohols and phenolic groups. The X-ray diffraction analysis showed various peaks ranging from 32.35 to 67.35°, and the highest intensity showed at 36.69°. The particle size ranged from 26 to 40 nm and confirmed the average particle size is 30.9 nm. The green synthesized *P. guajava* cobalt oxide nanoparticles contain cobalt as the major abundant element, with 42.26 wt% and 18.75 at% confirmed by the EDAX techniques. SEM images of green synthesized *P. guajava* cobalt oxide nanoparticles showed agglomerated and non-uniform spherical particles. The anti-bacterial activity of green synthesized *P. guajava* cobalt oxide nanoparticles was evaluated against Gram-positive *Staphylococcus aureus* and Gram-negative *Escherichia coli* with a 7 to 18 mm inhibitory zone. The photocatalytic activity was evaluated using green synthesized *P. guajava* cobalt oxide nanoparticles and observed 79% of dye degradation. The MTT assay of *P. guajava* cobalt oxide nanoparticles showed an excellent cytotoxic effect against MCF 7 and HCT 116 cells compared to normal cells. The percentage of cell viability of *P. guajava* cobalt oxide nanoparticles was observed as 90, 83, 77, 68, 61, 58 and 52% for MCF-7 cells and 82, 70, 63, 51, 43, 40, and 37% for HCT 116 cells at the concentration of 1.53, 3.06, 6.12, 12.24, 24.48, 50, and 100 μg/mL compared to control cells. These results confirmed that green synthesized *P*. *guajava* cobalt oxide nanoparticles have a potential photocatalytic and anti-bacterial activity and also reduced cell viability against MCF-7 breast cancer and HCT 116 colorectal cancer cells.

## 1. Introduction

Nanotechnology is essential for nanomaterial synthesis and development. Nanomaterials which have grown in popularity as an outcome of their minute size (1–100 nm) [1,2]. The two primary approaches for synthesizing nanomaterials are chemical and green synthesis [2,3]. Due to their vast and reactive surface area and small size, metallic nanoparticles (NPs) have remarkable physiochemical properties [4]. Various earth-metal oxide nanoparticles have been employed as electrode resources for electrochemical measurements, including manganese oxide (MnO_2_) [5], cobalt oxide (CoO) [6], and nickel oxide (NiO) [7]. Because of their dual activity, CoO nanoparticles and their composites are identified as effective catalysts for water oxidation and light absorbers that promote a wide-band-gap [8]. CoO, Co_2_O_3_, and Co_3_O_4_ are some of the potential oxidation states of cobalt, of which Cobalt oxide as a non-stoichiometric polymorph has gotten significant concentration due to its structural and redox features [9].

Cobalt oxide (Co_3_O_4_) nanoparticles are transition metal oxide nanoparticles with variable oxidation states [10] that are used in a variety of applications such as gas sensors, pigments, catalysts, magnetic materials, electronic devices, anode materials for rechargeable batteries, high-temperature solar selective absorbers and solar energy system magnetic devices and biological applications, such as cancer treatments [11,12,13,14]. Cobalt oxide catalyst’s high activity and selectivity are assumed to be due to variations in oxygen holes, oxygen adsorbed, and oxygen defects in different states of cobalt in Cobalt oxide and mixed valences [15,16]. Co_3_O_4_ NPs have a conventional cubic spinel crystal structure, with one-third of the cobalt atoms in the oxidation state of Co (II) occupying tetrahedral sites and the remaining two-thirds in the oxidation state of Co (III) occupying octahedral sites [17,18,19].

Water pollution is the most critical concern worldwide [20]. In recent years, water has been polluted and become hazardous due to various industries. such as textile and leather industries. Twenty percent of harmful dyes are usually discharged into water surfaces by textile factories, and it acts as carcinogenic in humans [20,21]. Direct Blue 71, an azo dye generally used in fabric industries, acts as a coloring agent and demonstrates a bright blue color [21]. Many dyes are used in industries without proper management, which affects the environment. Generally, the dye degraded by different methods such as ion exchange [22], electro-coagulation [23], redox treatment, and photodegradation [24,25]. Among all these degradation processes, scientists focused on degrading dyes using photo-catalytic activity.

Numerous methods have been established for the synthesis of cobalt oxide nanoparticles, such as precipitation, sol-gel, wet chemical, microwave-assisted, electrochemical, hydrothermal, reverse micelles and green synthesis [26,27,28,29]. Biosynthesis of NPs has become a prominent nanotechnology development. The physical and chemical methodologies used to synthesize nanoparticles are tremendously expensive and use hazardous chemicals [30]. There is now a lot of interest in using green chemistry to synthesize cobalt oxide nanoparticles using plant extract as a source. Plant extracts contain bioactive chemicals such as polyphenols, flavonoids and other glycosides, which act as a reducing agent by converting metal ions to NPs [31,32,33]. The production of cobalt oxide NPs using *Calotropis procera* latex was described by Dubey et al. [34]. Bibi et al. [35] made cobalt oxide NPs from cobalt nitrate hexahydrate using *Punica granatum* peel extract. Diallo et al. [36] exposed that the extract from *Aspalathus linearis* leaves can be used as a bio-reduction agent to produce cobalt oxide NPs.

*Psidium guajava*, a common name called guava, belongs to the Myrtaceae family, and it has a well-known tree with edible fruits [37,38,39]. Guava leaf aqueous extract contains flavonoids, alkaloids, glycosides, polysaccharides, terpenoids, steroids and saponins [40,41,42,43]. These leaves mainly contain quercetin, which includes antimicrobial, antioxidant, anticancer, anti-inflammatory and antiviral activity [43,44,45,46,47].

In this present study, we aimed to synthesize Co_3_O_4_ NPs from *P. guajava* aqueous extracts, which act as a stabilizing and reducing agent. The synthesized nanoparticles were further characterized by Fourier-transform infrared spectroscopy (FTIR analysis), X-ray diffraction analysis (XRD analysis), scanning electron microscopy (SEM analysis) and energy dispersive spectroscopy (EDAX) analysis. Further, the main objective of this study is to investigate the anti-bacterial, photocatalytic activity and effectiveness of green synthesized *P. guajava* Co_3_O_4_ NPs on MCF-7 and HCT 116 cells.

## 2. Results and Discussion

### 2.1. Spectroscopic Analysis

#### 2.1.1. UV-Visible Analysis

**Figure 1:**
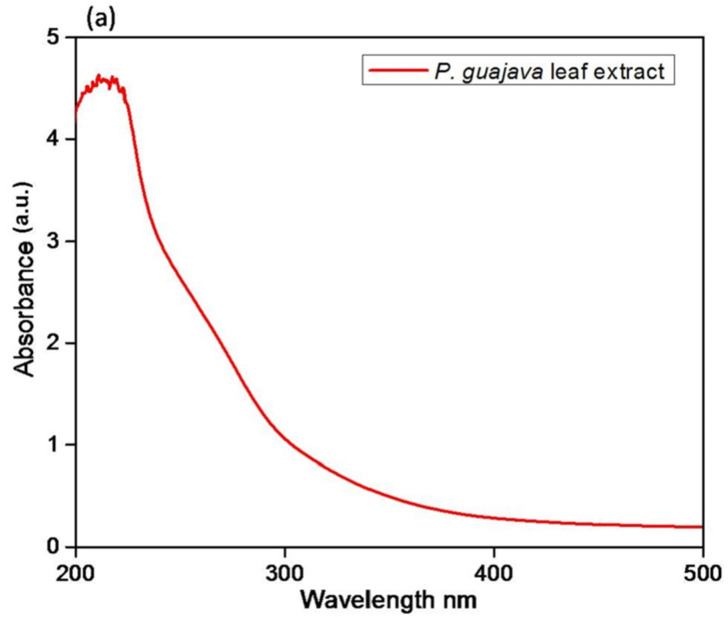

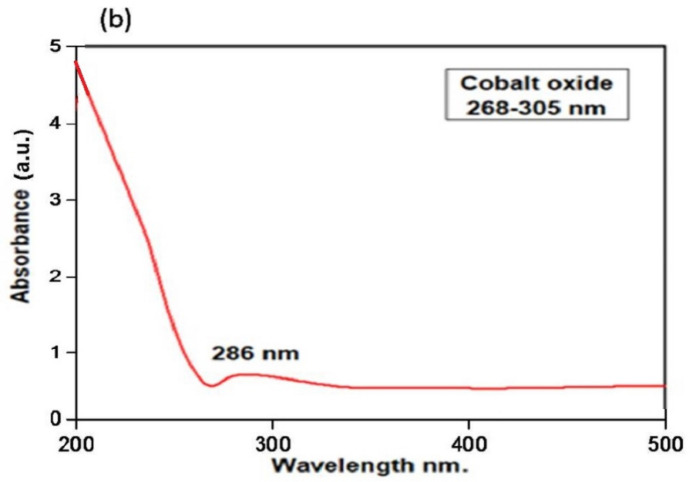
(**a**) UV–Visible spectrum of *Psidium guajava* leaf extract and (**b**) Green synthesized *P. guajava* Co_3_O_4_ NPs.

The synthesis of *P. guajava* Co_3_O_4_ NPs was initially identified through a shift in color of the reaction mixture from light brown to dark brown in 30 min at 37 °C. UV–visible spectra were used to analyze the reaction process between *P. guajava* leaf extract components and metal ions. The UV spectrum is a broad peak observed at 200–250 nm in *P. guajava* leaf extracts (Figure 1a). The UV spectrum of green synthesized *P. guajava* Co_3_O_4_ NPs exhibited an absorption band of 286 nm (Figure 1b) which confirms the nanoparticle synthesis.

The electrons continuously fluctuating in the conduction band generated through the binding of an electromagnetic field is the source of light absorption by metal NPs [48]. The plasmon resonance absorption of the *P. guajava* leaf extract is responsible for the obtained absorption band in the *P. guajava* Co_3_O_4_ NPs FTIR spectra. The particle size, dielectric medium, and surface-adsorbed species influenced plasmon absorption in cobalt nanoclusters [49]. The reduction of cobalt (II) nitrate hexahydrate in the existence of *P. guajava* leaf extract, which acts as a stabilizing, reducing and capping agent, resulted in cobalt oxide NPs [50]. The nucleation and growth processes are accomplished by reducing cobalt ions to neutral cobalt atoms. Another method is known as in situ green synthesis, in which the particles are initially synthesized by nucleation and then stabilized by the extract secondary metabolites [51].

#### 2.1.2. XRD Analysis

**Figure 2:**
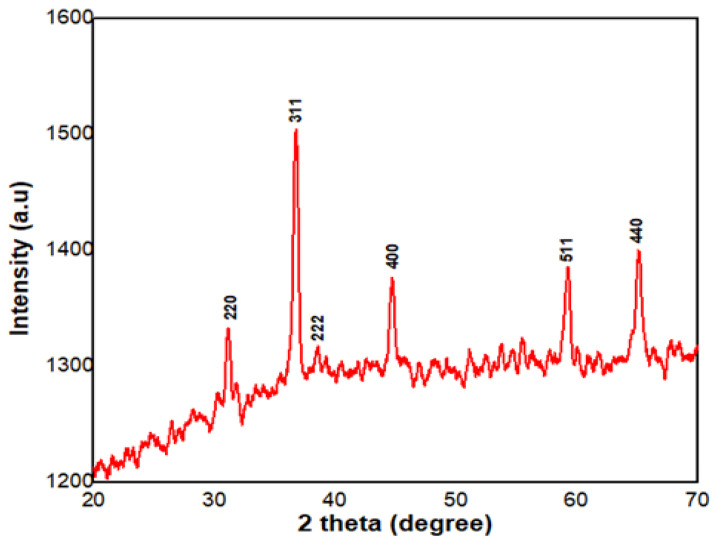
XRD analysis of *Psidium guajava* Co_3_O_4_ NPs (JCPDS Card No. 073-1701).

XRD analysis was used to determine the structural characteristics and crystalline nature of *P. guajava* Co_3_O_4_ NPs. Figure 2 depicts the XRD pattern of *P. guajava* Co_3_O_4_ NPs obtained from the *P. guajava* leaf extract. The observed intensity peaks at 32.35°, 36.69°, 39.24°, 44.76°, 59.42° and 67.35° are representing the corresponding planes at 220, 311, 222, 400, 511 and 440, respectively (Figure 2). The *P. guajava* Co_3_O_4_ NPs diffraction 2θ values correspond to the JCPDS No. 073-1701 standard database values [52,53].

The crystallite size of Co_3_O_4_ NPs, calculated by the Scherrer equation and based on the highest diffraction peak assigned to 311 crystal planes is about 36.6 positions. The X-ray diffraction analysis showed various peaks ranging from 32.35 to 67.35° and the highest intensity showed at 36.69° (Table 1). The particle size ranged from 26 to 40 nm and confirmed the average particle size is 30.9 nm.

#### 2.1.3. FTIR Analysis

**Figure 3:**
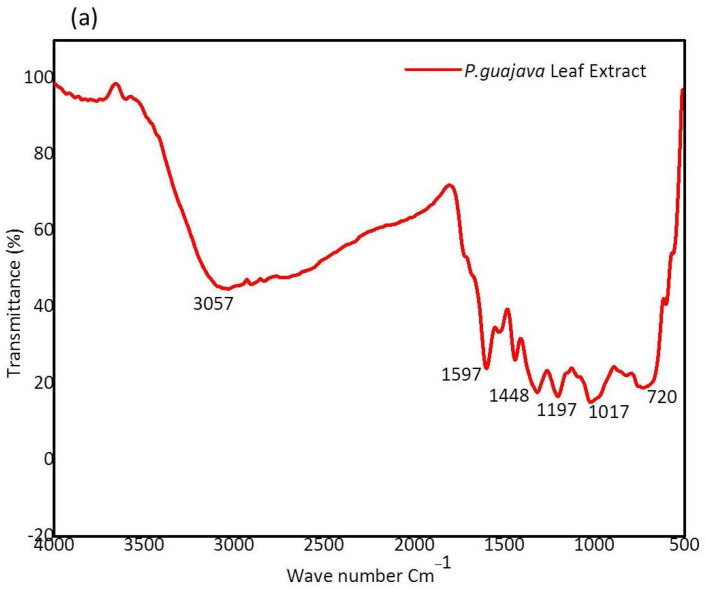

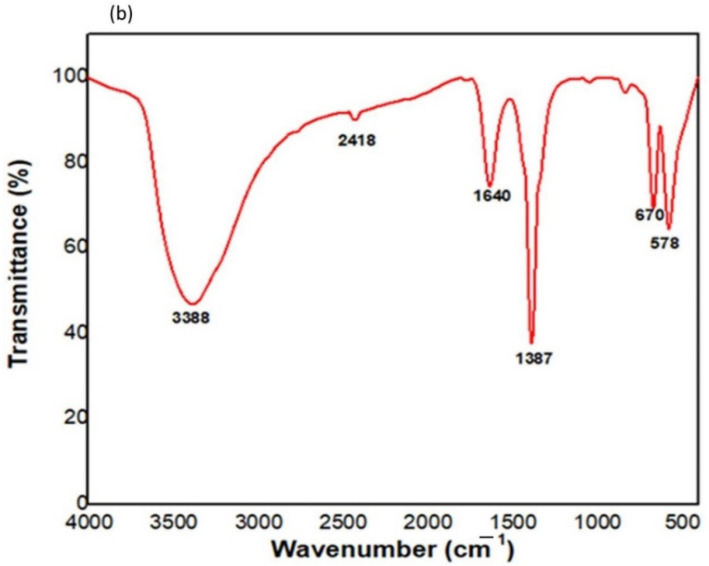
(**a**) FTIR spectrum of *Psidium guajava* leaf extract and (**b**) Green synthesized *P. guajava* Co_3_O_4_ NPs.

FTIR analysis was conducted to identify the various functional groups that are present in the *P. guajava* leaf extract and serve as capping and stabilizing agents. The FTIR spectrum of *P. guajava* leaf extract and green synthesized *P. guajava* cobalt oxide nanoparticles are shown in Figure 3a,b. The broad peak of *P. guajava* leaf extract at 3057 cm^−1^ corresponds to –C-H in aromatic and unsaturated hydrocarbons (=C-H stretch). A sharp peak observed at 1597 cm^−1^ represents NH_2_ in amino acids (NH_2_ deformation). IR spectrum of *P. guajava* leaf extract at 1448 cm^−1^ corresponds to -CH_3_ in aliphatic compounds (-CH_3_ antisym deformation) [53]. A sharp peak was noticed at 1197 cm^−1,^ which represents–C-OH in alcohols (C-O stretch). A medium peak was observed at 1017 cm^−1^ corresponding to –CH-OH in cyclic alcohols (C-O stretch). A broad peak was observed at 720 cm^−1,^ which represents –OH in phenols (-OH out of plane deformation).

The following IR spectrum of peaks observed in *P. guajava* CO_3_O_4_ NPs was noted at 3388 cm^−1^ corresponds to NH2 in aromatic amines, primary amines (-NH stretch). A medium peak noted at 2418 cm^−1^ represents –PH in phosphines (-PH stretch). The sharp peaks were observed at 1640 and 1387 cm^−1,^ which represents C=O in Benzophenones (C=O stretch) and SO_2_ in sulfonyl chlorides (SO_2_ antisym stretch).

### 2.2. Morphological Analysis

#### 2.2.1. Scanning Electron Microscopy (SEM)

**Figure 4:**
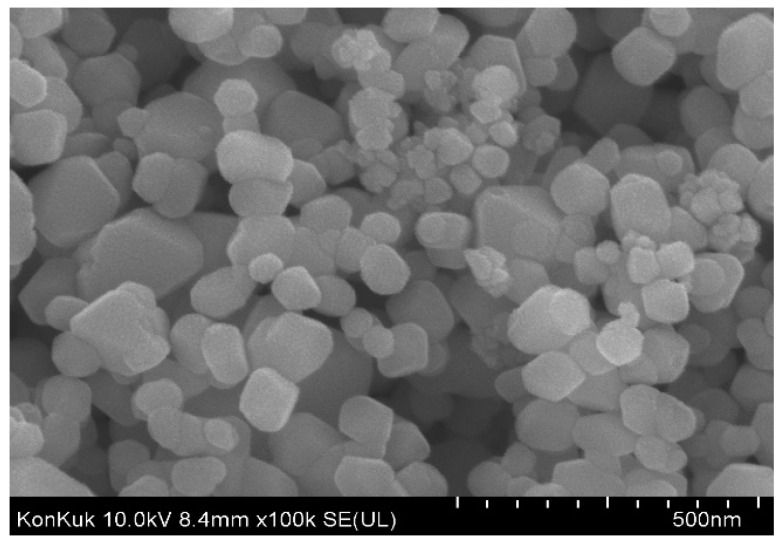
SEM image of *Psidium* *guajava* Co_3_O_4_ NPs.

SEM was used to analyse the surface morphology of *P. guajava* Co_3_O_4_ NPs, and the result is presented in Figure 4. The secondary metabolites and the chemical components present in *P. guajava* leaf extract influence particle agglomeration [54] because bioactive molecules enclose and stabilize each particle. Larger particles are formed due to the functional groups’ reactivity and attraction. These particles are coated with hydroxyl groups on the surface of various biological substances. The constituent part emerges to be agglomerated due to the formation of hydrogen bonding with the molecules surrounded by these agents [55]. It is clear from the SEM image that the constituent parts were non-uniform sphere-shaped, agglomerated and of an average size of 30.9 nm. As a result, the *P. guajava* Co_3_O_4_ NPs ability to suppress microorganisms is improved by their smooth surface.

#### 2.2.2. Energy Dispersive X-ray Analysis (EDAX)

**Figure 5:**
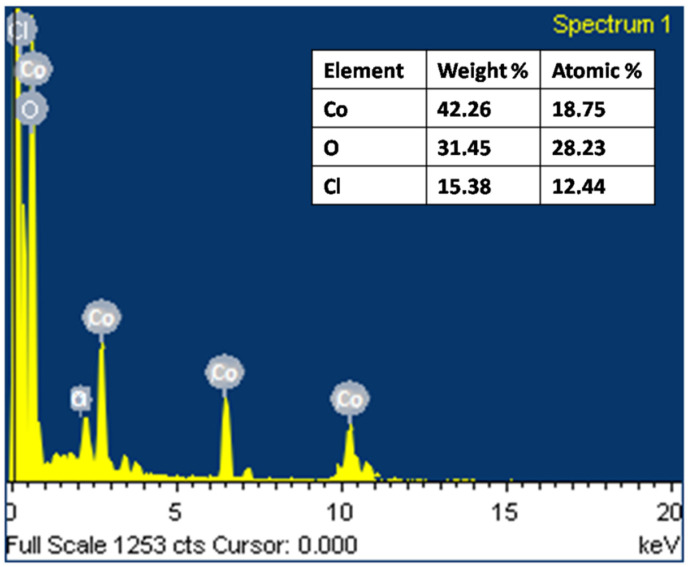
EDAX analysis of *Psidium guajava* Co_3_O_4_ nanoparticles.

The elemental composition of the synthesized *P. guajava* Co_3_O_4_ NPs was examined using EDAX at energies ranging from 0 to 10 keV based on their weight and atomic percentage. The spectra (Figure 5) demonstrated well-built peaks of cobalt in the prepared sample. A minor peak of carbon is present, which might be attributed to the *P. guajava* leaf extract [56]. The green synthesized *P.* *guajava* Co_3_O_4_ NPs elemental makeup indicates cobalt as a major element with 42.26 wt% and 18.75% at%. Co_3_O_4_ NPs contain oxygen with 31.45 wt% and 28.23 at%. The composition data from the EDAX spectra correlated well with the theoretically estimated values, implying that the nanoparticles have acceptable compositional homogeneity. The spectra showed sharp peaks between 0 and 1 KeV and between 6.5 and 8 KeV.

### 2.3. Photocatalytic Activity

**Figure 6:**
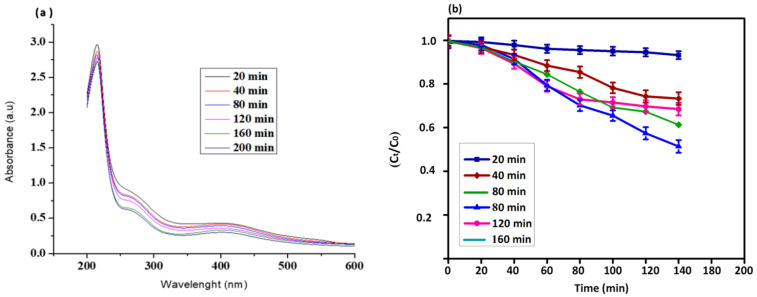
(**a**) UV-visible analysis (**b**) Dye degradation using *Psidium guajava* Co_3_O_4_ NPs as a catalyst under solar light irradiation.

The photocatalytic degradation of *P. guajava* Co_3_O_4_ NPs were studied under solar light irradiation and were confirmed for dye degradation of direct blue 71 under UV light. The absorption peak at 591 nm noticed the dye degradation in the presence of Co_3_O_4_ NPs. About 79% of the dye degradation was observed in 80 min using NPs. The UV-visible spectrum of direct blue dye degradation is shown in Figure 6a. The reduction in the absorbance represents the capability of *P. guajava* Co_3_O_4_ NPs to degrade direct blue dye. The percent of dye degradation reaction shown in Figure 6b was also deliberated by plotting ln (Ct/C_0_) aligned with the time (min) of the reaction.

The dye band reduced as the reaction time increased, and a new peak emerged, indicating that the dye was destroyed through photocatalytic treatment [57]. The chromophoric group’s degradation and the dye’s formation into low molecular weight by-products are connected with the photocatalytic degradation of the target dye. Under irradiation, dye degradation is primarily caused by creating electrons and holes (e^−^ & h^+^) on the catalyst surface [58]. The water molecule combines with the hole, which is formed by the dye degradation and gets converted to OH radical, whereas the O_2_ scavenges the e^-^ and renovates it into OH through HOO and H_2_O_2_ intermediate. The OH is a powerful oxidizing agent that degrades the dye into the water, carbon-di-oxide and inorganic ions non-selectively [59].

### 2.4. Antioxidant Activity

#### 2.4.1. DPPH Assay

**Figure 7:**
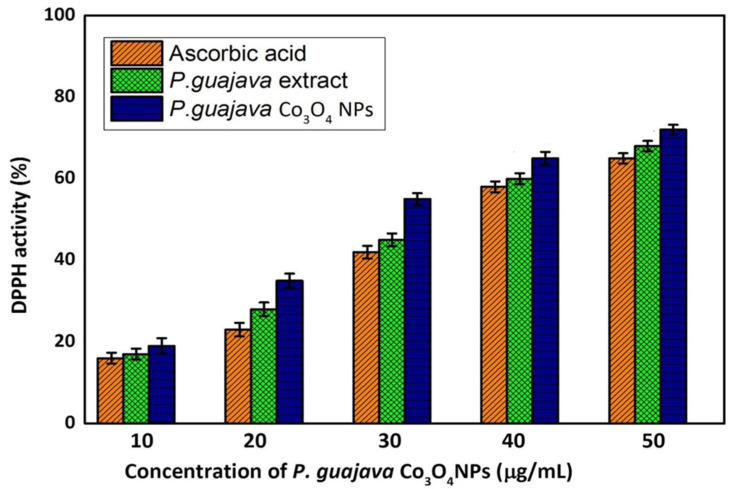
Antioxidant activity of *Psidium guajava* Co_3_O_4_ NPs using DPPH assay. Results are represented as mean ± SD, triplicate measures.

Flavonoid and polyphenolic constituents in *P. guajava* leaves extracts have antioxidant activity, preventing cells from oxidative stress caused by the free radicals [60]. Free radical scavenging activities are vital for avoiding the harmful effects of various illnesses, including cancer. The antioxidant properties of plant extracts were usually examined using the DPPH assay. The ability of antioxidants to donate hydrogen is understood to be the reason for their effect on DPPH [61]. The compounds extracted into a violet DPPH solution are reduced to a yellow color, based on concentration. The DPPH free radical scavenging activity of the *P. guajava* Co_3_O_4_ NPs was depicted in Figure 7.

The DDPH results indicated significant DPPH activity at a 50 μg/mL concentration of *P. guajava* Co_3_O_4_ NPs. However, 67% of activity exhibited at 50 μg/mL concentration compared to the standard ascorbic acid. The leaf extracts of *P. guajava* with high total phenolic and flavonoid content showed strong DPPH radical-scavenging activity because they exhibited a high hydrogen-donating capability to scavenge DPPH radicals. The current study had similar results to those reported by Jahani et al. [62]. An increase in phenolic and flavonoid contents leads to their propensity to donate electrons to scavenge DPPH radicals, resulting in substantial DPPH radical scavenging activity, which explains that the DPPH radical-scavenging activity being related to the content of phenolic and flavonoid components in the extracts. It may be attributed due to the fact that *P. guajava* Co_3_O_4_ NPs act as a good oxidant. Moreover, *P. guajava* leaves extracts contain secondary metabolites, flavonoids and phenolic compounds and the synthesized *P. guajava* Co_3_O_4_ NPs act as an excellent antioxidant activity.

#### 2.4.2. ABTS Assay

**Figure 8:**
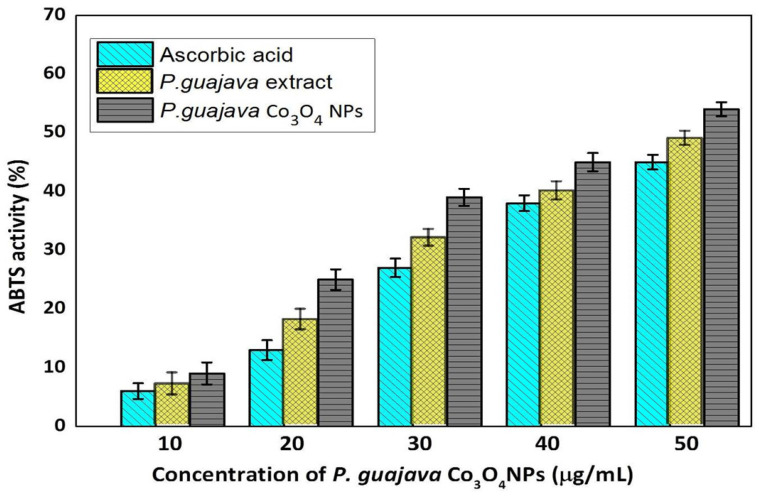
Antioxidant activity of *Psidium guajava* Co_3_O_4_ NPs using ABTS. Results are represented as mean ± SD, triplicate measures.

Free radicals are created constantly in the human body and become stable by releasing electron or hydrogen radicals, resulting in the breakdown of targets e.g., lipids, DNA and proteins, which are involved in the formation of diseases such as cardiovascular disorders, cancer and neurodegenerative diseases [63]. The outcome of the ABTS free radical scavenging activity of the *P. guajava* Co_3_O_4_ NPs was depicted in Figure 8.

The ABTS results indicated the significant scavenging activity at a 50 μg/mL concentration of *P. guajava* Co_3_O_4_ NPs. However, 54.5% of activity exhibited at 50 μg/mL concentration compared to the standard Ascorbic acid. The ABTS radical scavenging activity was observed to increase as the dose of *P. guajava* Co_3_O_4_ NPs was increased, when compared to the *P. guajava* leaf extract. This could be attributed to the 50 μg/mL concentration of NPs that can effectively interact with the ABTS free radicals [63].

### 2.5. Anti-Bacterial Activity

**Figure 9:**
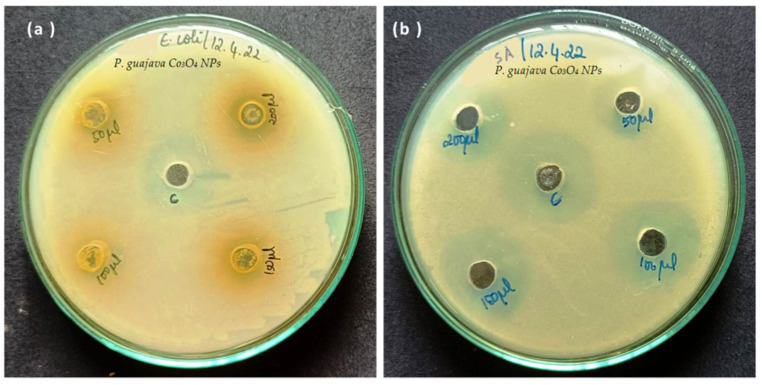
Anti-bacterial activity of green synthesized *Psidium guajava* Co_3_O_4_ NPs against (**a**) gram-negative bacteria (*E. coli*) (**b**) gram-positive (*S.* *aureus*).

The anti-bacterial activity was determined using the Agar well diffusion technique. Gram-positive and gram-negative bacteria were evaluated against the green synthesized *P*. *guajava* Co_3_O_4_ NPs and control used dimethyl sulfoxide (DMSO). The *P. guajava* Co_3_O_4_ NPs exhibited good anti-bacterial activity against tested bacterial strains. The zone of inhibition of *P. guajava* Co_3_O_4_ NPs was observed as 9, 12, 16, and 18 mm for *Staphylococcus aureus* (gram-positive) bacteria. The zone of inhibition of *P. guajava* Co_3_O_4_ NPs was observed as 7, 10, 13 and 15 mm for *E. coli* (gram-negative) bacteria (Table 2). Figure 9a,b showed that *P. guajava* Co_3_O_4_ NPs have excellent anti-bacterial activity against gram-positive (*S. aureus*) bacteria than gram-negative (*E. coli*). The smaller nanoparticle size leads to greater antimicrobial activity. In previous literature, Bose et al. [51] investigated the surface behavior of nanoparticles [64,65]. The surface of the green synthesized *P. guajava* Co_3_O_4_ NPs is very smooth, and these facilities are used to interact with the microorganism’s cell wall.

### 2.6. MTT Assay of Vero, MCF-7 and HCT 116 Cells

**Figure 10:**
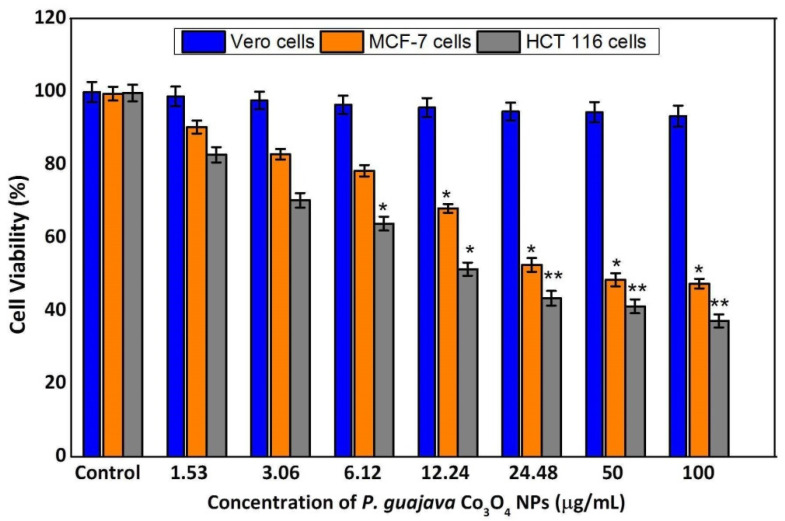
Cytotoxicity assay of green synthesized *Psidium guajava* Co_3_O_4_ NPs against Vero cells, MCF-7 cells, and HCT 116 cells. *P. guajava* Co_3_O_4_ NPs reduced the cell viability in a dose-dependent manner. Vero cells, MCF-7 and HCT 116 cells were treated with *P. guajava* Co_3_O_4_ NPs at different concentration (1.53, 3.06, 6.12, 12.24, 24.48, 50 and 100 μg/mL) for 24 h. Results are represented as mean ± SD, triplicate measures. * *p* < 0.05, ** *p* < 0.01.

The NPs incorporated into the biological circulation based on NPs physiochemical characteristics. The migration of NPs to distant regions is the major problem in their potential cytotoxicity [66]. Therefore, in this study, we evaluated to determine the cytotoxic efficacy of green synthesized *P. guajava* Co_3_O_4_ NPs against breast and colorectal cancer cell lines by MTT assay. Figure 10 showed the dose-dependent (1.53, 3.06, 6.12, 12.24, 24.48, 50 and 100 μg/mL) cytotoxic effect against Vero, MCF-7 and HCT 116 cell lines. Following 24 h incubation, there is no significant cytotoxic activity in Vero cells. The percentage of cytotoxic activity of green synthesized *P. guajava* Co_3_O_4_ NPs was observed as 90%, 83%, 77%, 68%, 61%, 58% and 52% for MCF-7 cells and 82%, 70%, 63%, 51%, 43%, 40%, and 37% for HCT 116 cells compared to control cells. The cell viability was decreased with increasing concentrations of green synthesized *P. guajava* Co_3_O_4_ NPs. The MTT assay revealed the significant dose-dependent cytotoxicity induced by *P. guajava* Co_3_O_4_ NPs. These findings are in accordance with the cytotoxic impact of these NPs, as previously reported [66]. The half maximal inhibitory concentration (IC_50_ value) was observed as 24.5 μg/mL for HCT 116 cells and 29.5 μg/mL for MCF-7 cells. These results revealed that *P. guajava* Co_3_O_4_ NPs has high cytotoxic effect in HCT 116 colorectal cancer cells than the MCF-7 breast cancer cells.

## 3. Materials and Methods

### 3.1. Chemicals

Cobalt oxide (Co_3_O_4_), cobalt nitrate (Co(NO_3_)_2_.xH_2_O), Dimethyl sulfoxide (DMSO), DPPH (2,2-diphenyl-1-picrylhydrazyl), ABTS (2,2′-azino-bis (3-ethyl benzothiazoline-6-sulfonic acid)), potassium persulfate, methanol, Muller Hinton agar medium, Direct Blue 71 dye, MTT (3-(4,5-Dimethylthiazol-2-yl)-2,5-Diphenyltetrazolium Bromide), Fetal bovine serum (FBS), Dulbecco’s Modified Eagle Medium (DMEM) were procured from Sigma Aldrich (St. Louis, MO, USA).

### 3.2. Preparation of Leaves Extract

The *Psidium guajava* leaves extracts were collected from Vellore, Tamil Nadu. The collected leaves were chopped and dried for 4 days in the shade. After that, the dried leaves extract was finely grounded until to obtain the powder form. Further, 12 g of powdered leaves extract were added to 100 mL of double-distilled deionized water. Then, the solution containing the beaker was kept at 60 °C for 5 h using a magnetic stirrer. The derived extract was called *P. guajava* aqueous extracts [60]. These extracts are used for the synthesis of Co_3_O_4_ NPs.

### 3.3. Biosynthesis of Co_3_O_4_ NPs

10 mL of the *P. guajava* leaf extract was added to 6 g of cobalt nitrate solution to synthesize cobalt oxide (Co_3_O_4_) nanoparticles [67]. This reaction solution was kept on the hot plate for 3 h to synthesize cobalt nanoparticles. After 3 h, the solution was transferred into the oven at 100 °C for 5 h to get the dried precipitate. Further, the precipitate cobalt oxide NPs were calcinated at 500 °C for 3 h. Finally, synthesized Co_3_O_4_ NPs was characterized by spectroscopic and microscopic analysis.

### 3.4. Characterization

*P. guajava* Co_3_O_4_ NPs were characterized using UV-Visible spectroscopy (UV-Vis), Fourier-transform infrared spectroscopy (FTIR analysis-Therma Corp., Nicollet, MI, USA), Powder XRD analysis (XRD analysis using Siemens 850, Borken, Germany), Scanning electron microscopy (SEM analysis Zeiss EVO LS10, Rugby, UK), Energy dispersive spectroscopy (EDAX) analysis [34].

### 3.5. Photocatalytic Activity

The photocatalytic activity of synthesized *P. guajava* Co_3_O_4_ NPs was studied for direct blue 71 dye degradation. 100 mL of 20 ppm direct blue 71 solutions were taken in a 500 mL beaker, and then 20 mg of *P. guajava* Co_3_O_4_ NPs were added and stirred for 60 min in dark conditions. Direct Blue 71 dye photo-degradation was carried out using a photoreactor under UV light at 365 nm [58]. 3 mL of direct blue dye solution was withdrawn at regular time intervals (20 min) for UV analysis (Shimadzu UV 1800, Torrance, CA, USA). The percentage of degradation was calculated below the equation.

(1) $$Degradation\;(\%)=\frac{C_{o}-C_{t}}{C_{o}}\;\times \;100$$

Here *C_o_* is the initial dye concentration, and *C_t_* is a concentration of dye after degradation at various times (*t*).

### 3.6. Antioxidant Activity

#### 3.6.1. DPPH Assay

An equal volume of *P. guajava* Co_3_O_4_ NPs of various concentrations (20–100 μg/mL) was mixed with DPPH (2,2-diphenyl-1-picrylhydrazyl) containing methanol solution. The nanoparticles were mixed with DPPH solution and kept for 60 min in a dark place. After 60 min of incubation, the reduction of DPPH radicals was monitored by UV-Spectrophotometer (Shimadzu UV 1800, Torrance, CA, USA) absorption at 517 nm [62]. The following formula was used to calculate the percentage inhibition of DPPH scavenges.

$$DPPHscavengingactivity\left(\%\right)=\frac{\left(A_{o}-A_{1}\right)}{A_{o}}\times 100\;A_{1}=\mathrm{absorbance}\;\mathrm{of}\;\mathrm{sample}\;A_{o}=\mathrm{absorbance}\;\mathrm{of}\;\mathrm{control}$$

#### 3.6.2. ABTS Scavenging Activity

The stock solution was prepared using 7 mM ABTS (2,2′-azino-bis (3-ethyl benzothiazoline-6-sulfonic acid) solution and 2.4 mM potassium persulfate solution. The working solution was then made by mixing the two stock solutions in equal amounts OF *P. guajava* Co_3_O_4_ NPs and allowing them to react for 14 h in the dark at room temperature. Using a spectrophotometer (Shimadzu UV 1800, Torrance, CA, USA), the solution was diluted by mixing 1 mL ABTS solution with 60 mL methanol to yield an absorbance of 0.706, 0.01 units at 734 nm [63]. The following formula used to calculate the percentage inhibition of ABTS scavenging activity is

(2) $$ABTS\;scavenging\;activity\left(\%\right)=\frac{\left(A_{o}-A_{1}\right)}{A_{o}}\times 100\;A_{1}=\mathrm{absorbance}\;\mathrm{of}\;\mathrm{sample}\;A_{o}=\mathrm{absorbance}\;\mathrm{of}\;\mathrm{control}$$

### 3.7. Anti-Bacterial Activity

The anti-bacterial activity of green synthesized *P. guajava* Co_3_O_4_ NPs was analyzed by the agar well diffusion method. First, the nutrient agar was uniformly spread in the Petri plates. The two bacterial strains, gram-positive (*Staphylococcus aureus*) and gram-negative (*Escherichia coli*) bacteria were used to examine the anti-bacterial activity [51]. After, 50, 100, 150 and 200 μL of *P. guajava* Co_3_O_4_ NPs was added to the Petri plates. The culture medium was incubated at 37 °C for 24 h. Following 24 h incubation, the zone of inhibition was measured.

### 3.8. Cytotoxicity Assay

The cytotoxicity assay of green synthesized *P. guajava* Co_3_O_4_ NPs was performed using the previously reported method [68]. Vero, MCF-7 and HCT 116 cell lines were plated in 96 well plates with 1 × 10^5^ cells/well in DMEM medium containing 10% Fetal bovine serum (FBS). The MCF-7 and HCT 116 cells exposed to *P. guajava* Co_3_O_4_ NPs at different concentration (1.53, 3.06, 6.12, 12.24, 24.48, 50 and 100 μg/mL) and incubated for 24 h. Following NPs exposure,10 μL of MTT (5 mg/mL) was added and kept in a CO_2_ incubator for 4 h. The supernatant was removed and 100 μL DMSO was added to each well. The optical density was measured by UV-Spectrophotometer (Shimadzu UV 1800, Torrance, CA, USA) absorption at 540 nm.

### 3.9. Statistical Analysis

The data were expressed as mean ± standard deviation. Statistical analysis of the data was performed by one-way analysis of variance (ANOVA) followed by Tukey’s multiple range tests for post-hoc analysis using the Sigmastat Version 3.5 software (Systat Software Inc., San Jose, CA, USA). The significance level was set at * *p* < 0.05, * *p* < 0.01.

## 4. Conclusions

Nanoscience and nanobiotechnology have grown in significance in the field of cancer treatment in order to optimize therapeutic effectiveness and minimizing cellular toxicity. Numerous therapies, such as chemotherapy, radiation, and many others, have been used in cancer treatment until now to completely eradicate cancer cells. The results from these approaches are unsatisfactory since any cells that are left over continue to divide and develop into new cells. To solve these concerns, nanotechnology and nanoparticles have been extensively utilized. Cobalt oxide nanoparticles were synthesized using *P. guajava* aqueous extract. The synthesized *P. guajava* Co_3_O_4_ NPs were characterized using UV-visible spectroscopy, FTIR analysis, XRD, SEM, and EDAX analysis. The primary confirmation of UV–Visible analysis exhibits a strong absorption peak at 286 nm. The FTIR analysis confirmed the functional groups alcohol and amide group from synthesize *P. guajava* Co_3_O_4_ NPs. Green synthesized *P. guajava* Co_3_O_4_ NPs were discovered by XRD examination to be pure crystalline in nature. SEM-EDAX characterization exhibited an agglomerated spherical morphology of synthesized *P. guajava* Co_3_O_4_ NPs. The *P. guajava* Co_3_O_4_ NPs exhibited high antioxidant DPPH and ABTS scavenging activities. The *P. guajava* Co_3_O_4_ NPs was found to be active photocatalysts used for the degradation of direct blue 71. The MTT assay revealed the significant dose-dependent cytotoxicity induced by *P. guajava* Co_3_O_4_ NPs. The half maximal inhibitory concentration (IC_50_ value) was observed as 24.5 μg/mL for HCT 116 cells and 29.5 μg/mL for MCF-7 cells. These results revealed that *P. guajava* Co_3_O_4_ NPs has high cytotoxic effect in HCT 116 colorectal cancer cells than the MCF-7 breast cancer cells, which suggests that *P. guajava* Co_3_O_4_ NPs may be utilized for a wide range of cancer treatments.

## Figures and Tables

**Table 1:** The peak positions of X-ray Diffraction of *Psidium guajava* Co_3_O_4_ NPs.

Peak Position 2θ (Degree)	FWHM β (Degree)	Crystallite Size (nm)
32.35	0.321	26.92
36.69	0.373	23.45
39.24	0.268	32.28
44.76	0.241	37.25
59.42	0.315	30.24
67.35	0.282	35.38
Average crystallite size (nm)		30.92 nm

Abbreviation: FWHM—Full-Width Half Maximum.

**Table 2:** Zone of inhibition of green synthesized *Psidium guajava* Co_3_O_4_ NPs against gram +ve (*Staphylococcus aureus*) and gram −ve (*Escherichia coli*) bacteria. Results are represented as mean ± SD, triplicate measures.

Concentration (µg/mL)	Zone of Inhibition (mm) *S. aureus*	*E. coli*
50	9 ± 1.25	7 ± 0.95
100	12 ± 1.97	10 ± 1.83
150	16 ± 2.45	13 ± 2.08
200	18 ± 2.69	15 ± 2.17

## Data Availability

Not available.
